# Only Half Right: Species with Female-Biased Sexual Size Dimorphism Consistently Break Rensch's Rule

**DOI:** 10.1371/journal.pone.0000897

**Published:** 2007-09-19

**Authors:** Thomas J. Webb, Robert P. Freckleton

**Affiliations:** Department of Animal and Plant Sciences, University of Sheffield, Sheffield, United Kingdom; Zoological Society of London, United Kingdom

## Abstract

**Background:**

Most animal species display Sexual Size Dimorphism (SSD): males and females consistently attain different sizes, most frequently with females being larger than males. However the selective mechanisms driving patterns of SSD remain controversial. ‘Rensch's rule’ proposes a general scaling phenomenon for all taxa, whereby SSD increases with average body size when males are larger than females, and decreases with body size when females are larger than males. Rensch's rule appears to be general in the former case, but there is little evidence for the rule when females are larger then males.

**Methodology/Principal Findings:**

Using comprehensive data for 1291 species of birds across 30 families, we find strong support for Rensch's rule in families where males are typically larger than females, but no overall support for the rule in families with female-biased SSD. Reviewing previous studies of a broad range of taxa (arthropods, reptiles, fish and birds) showing predominantly female-biased SSD, we conclude that Rensch's conjecture is the exception rather than the rule in such species.

**Conclusions/Significance:**

The absence of consistent scaling of SSD in taxa with female-biased SSD, the most prevalent direction of dimorphism, calls into question previous general evolutionary explanations for Rensch's rule. We propose that, unlike several other ecological scaling relationships, Rensch's rule does not exist as an independent scaling phenomenon.

## Introduction

Most species of animal display Sexual Size Dimorphism (SSD): males and females consistently attain different sizes [1]–[4]. However, the selective mechanisms driving patterns of SSD remain controversial. Sexual selection is frequently invoked [5], but interactions between sexual selection, viability selection and fecundity selection are likely to be important in determining the precise form of SSD in any particular case [6]. Intersexual differences in adult body size are necessarily mediated by variation in organism growth and development [7], and so uncovering the evolutionary mechanisms responsible for interspecific patterns of SSD will also have an important bearing on understanding the evolution of life histories. The search for generalities in patterns of SSD across species therefore continues to receive considerable attention [2], [3], [6].

The pattern most commonly cited in studies of SSD has become known as Rensch's rule, which appeared in English in Rensch's 1959 book *Evolution above the species level* [8]:

“…the rule is valid that in numerous animal groups the sexual dimorphism increases with body size (B. Rensch, 1950). This rule, however, applies only to subspecies of a species, to related species of a genus, or to related genera of a family. In species of birds in which the male is larger than the female, the relative sexual difference increases with body size. If, by way of exception, the females are larger than the males, as among many species of birds of prey, the opposite correlation applies, i.e. the greater sexual difference is found in the smaller species.” (p 157–158)

Rensch's first conjecture has received widespread support from studies of diverse vertebrate and invertebrate taxa: in taxa where males are larger than females (male-biased SSD, MBSSD), SSD does indeed tend to increase with body size [2], [3]. In taxa with female-biased SSD (FBSSD), however, evidence for Rensch's rule (i.e., that SSD should decrease with body size) is extremely scarce. For instance, across 7 taxonomic groups with FBSSD reviewed in [2], evidence for and against Rensch's rule was almost exactly balanced. Indeed, as shown in [9], the data from which Rensch drew his original conclusion showed no evidence for smaller FBSSD species to be more dimorphic than larger FBSSD species. Thus, although in certain taxa with FBSSD scaling of dimorphism consistent with Rensch's rule does occur, this pattern lacks generality and so presents a serious barrier to the acceptance of Rensch's rule as a comprehensive explanation of SSD–particularly as FBSSD predominates in the animal kingdom [2].

**Figure 1:**
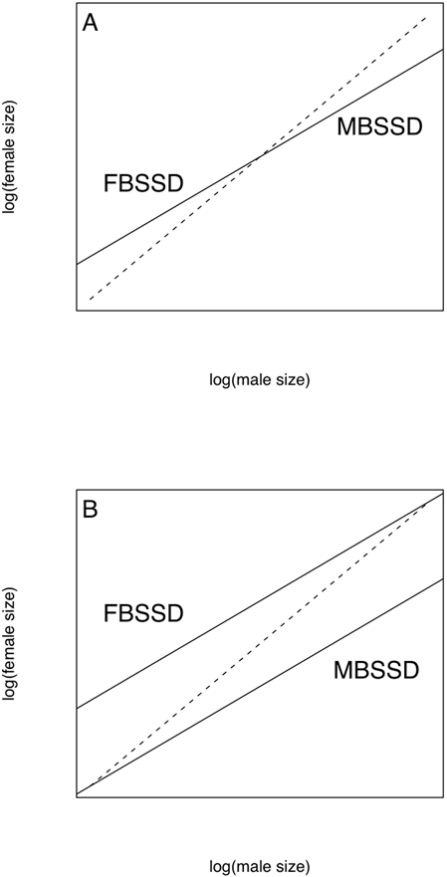
A. Rensch's rule as it is often represented schematically. The dashed line is the line of isometry (male size = female size); the solid line represents Rensch's rule, with SSD increasing with size in species with MBSSD, decreasing with size in species with FBSSD. However, this representation also makes the assumption (not stated by Rensch) that in taxa with mixed SSD, those species with FBSSD will be smaller than species with MBSSD. B. An alternative representation of Rensch's rule, with SSD decreasing with size in FBSSD species, increasing in MBSSD species as before, but both MBSSD and FBSSD species can span the full range of sizes present in the group.

The usual way to test for scaling of SSD consistent with Rensch's rule is to test whether the slope of the relationship between female and male size (both log-transformed) differs significantly from 1 [2], [3], [10]. When male size is the independent variable and female size the dependent, a slope significantly less than 1 is taken as evidence in support of Rensch's rule, with SSD increasing with male size in species with MBSSD and decreasing with male size in species with FBSSD. Likewise SSD may be regressed on either male or female size with statistically identical results to the female-on-male or male-on-female regressions, but with a slope of 0 indicating isometry [10]. The pattern is often conceptualised as shown in figure 1A [2], [3], [11]–[13].

This representation of Rensch's rule has been taken to imply that in taxa which display mixed SSD (i.e. some MBSSD, some FBSSD species), species with FBSSD will be smaller than those with MBSSD. This pattern is interesting in its own right, but is neither necessary for nor indicative of Rensch's rule. Rather, Rensch's rule is better characterised as shown in figure 1B [see also 14]. Here, the expectation for taxa with mixed SSD is that the slopes for species with MBSSD and species with FBSSD considered separately will both be <1. This approach has rarely been adopted, and indeed some studies of Rensch's rule in taxa with mixed SSD taxa explicitly state that what they test is that FBSSD species are smaller than MBSSD species [12], [15].

Many studies of SSD are based on measurements of very small numbers of species from very speciose groups [2], [6]. These studies provide useful indications of general patterns, but there is a pressing need for more comprehensive analyses incorporating a large sample taken from across all taxa within a clade. Birds provide an excellent model for such a study, as body sizes are available for very large numbers of species. In addition, birds are ecologically, behaviourally and morphologically diverse, displaying the full range of SSD. However, previous studies of SSD in birds have provided mixed support for Rensch's rule [2], [5], [16], [17], perhaps because they have been based on taxonomically restricted samples or limited numbers of species. The most comprehensive analysis to date [18] found general support for Rensch's rule in birds, but did not distinguish between taxa with MBSSD and FBSSD.

Here, we analyse a comprehensive dataset of male and female body mass estimates for 1291 species of birds to test the generality of Rensch's rule across this class. We test whether the existence of Rensch's rule within an avian family is dependent upon the proportion of species which display MBSSD. In particular, we assess whether Rensch's rule is observed in families with predominantly FBSSD. In families with mixed SSD, we also test whether scaling consistent with Rensch's rule is more commonly observed among the subset of species with MBSSD or that with FBSSD. In the light of our results, we then review studies of Rensch's rule in other taxa with FBSSD including arthropods, fish and reptiles. We discuss the important implications of our findings for hypotheses regarding the evolutionary mechanisms behind interspecific patterns in SSD.

## Methods

### Data

We compiled a dataset of male and female size (body mass, g) for 1520 species of birds. The data were largely obtained from various sources by I.P.F. Owens [see 19 for more details], but we supplemented this dataset with data from published studies examining Rensch's rule in hummingbirds [12], tinamous [17] and shorebirds [5] as these groups contain significant numbers of species with FBSSD. The data are drawn from across Class Aves, with 22 orders and 102 families represented. The full spectrum of avian SSD is also represented, from the great bustard *Otis tarda* (males 2.66×heavier than females) to the grey goshawk *Accipiter novaehollandiae* (females 2.13×heavier than males). Overall, MBSSD occurs in 932 species, FBSSD in 549 species, and there is no SSD in 39 species.

### Statistical Analyses

There has been extensive discussion in the literature of the appropriate statistical techniques for analysing allometry in SSD [reviewed in 10]. When performing a simple regression of log(female) on log(male) size, measurement error will be approximately equal in both sexes. The OLS estimate of the slope of the relationship will therefore be biased and many authors have preferred a Model II regression (e.g. Reduced Major Axis, RMA). However, the difference between OLS and RMA estimates approaches 0 as the correlation between male and female size approaches 1 (as *b*_OLS_ = *rb*_RMA_)[20], and high correlations (>0.9) are generally the case between male and female size (*r* = 0.996 for the 1520 species our dataset and generally >0.99 for each family (table 1); see also 3).

**Table 1:** Family specific slopes of log(female) on log(male) size (with 95% CIs) for the 30 families in our dataset with ≥10 species.

Family	No. species^a^	No. species with MBSSD (Proportion of all species)	Correlation (log(m) and log(f))	Slope^b^ (95% CI)
Falconidae	30	1 (0.03)	0.983	1.06 (1.00–1.13)
Strigidae	27	1 (0.04)	0.996	1.03 (0.99–1.08)
Acciptridae	104	4 (0.04)	0.990	0.94 (0.92–0.96)
Tinamidae	25	1 (0.04)	0.979	0.99 (0.91–1.07)
Scolopacidae	73	6 (0.08)	0.958	1.00 (0.97–1.04)
Muscicapidae	35	14 (0.40)	0.995	1.03 (0.97–1.08)
Charadriidae	62	27 (0.44)	0.998	0.98 (0.95–1.02)
Apodidae	12	6 (0.50)	0.950	0.99 (0.90–1.07)
Cuculidae	14	7 (0.50)	0.990	0.83 (0.72–0.94)
Funariidae	14	7 (0.50)	0.996	1.00 (0.89–1.10)
Caprimulgidae	11	6 (0.55)	0.989	0.92 (0.73–1.10)
Glareolidae	10	6 (0.60)	0.994	1.12 (0.95–1.29)
Psittacidae	32	20 (0.62)	0.992	0.96 (0.92–1.01)
Thamnophilidae	20	13 (0.65)	0.991	1.04 (0.95–1.13)
Trochilidae	159	109 (0.69)	0.980	0.86 (0.82–0.91)
Tyrannidae	67	49 (0.73)	0.994	0.96 (0.92–0.99)
Laridae	74	55 (0.74)	0.979	0.95 (0.92–0.98)
Columbidae	42	32 (0.76)	0.996	1.00 (0.95–1.05)
Passeridae	18	14 (0.78)	0.991	0.93 (0.64–1.21)
Fringillidae	142	113 (0.80)	0.994	0.90 (0.87–0.93)
Ardeidae	19	16 (0.84)	0.996	0.92 (0.86–0.98)
Rallidae	38	33 (0.87)	0.974	0.94 (0.91–0.98)
Picidae	48	43 (0.90)	0.995	0.97 (0.93–1.02)
Anatidae	77	70 (0.91)	0.988	0.98 (0.95–1.02)
Cracidae	11	10 (0.91)	0.995	0.93 (0.83–1.03)
Gruidae	11	10 (0.91)	0.959	0.90 (0.73–1.07)
Sylviidae	22	20 (0.91)	0.986	1.01 (0.92–1.09)
Odontophoridae	14	13 (0.93)	0.989	0.93 (0.76–1.11)
Corvidae	37	35 (0.95)	0.993	0.96 (0.95–1.00)
Phasianidae	43	41 (0.95)	0.997	0.88 (0.84–0.92)

a The total number of species is the number of species for which we had data on male and female size.

b This is the OLS slope, note that the RMA slope for a family can be obtained by dividing the OLS slope by the correlation coefficient.

To further validate this assumption that the method of analysis used was valid, we used a Simulation-Extrapolation approach (SIMEX)[21]. For two datasets for which we had estimates of measurement error (i.e. intraspecific variation in male size), we show that the OLS estimate is at least as good an approximation of the slope as the RMA estimate (see Text S1). We note however that all three estimates of slope (OLS, RMA and SIMEX; note the scale on the y-axis on Figure S1) are extremely similar, and we agree with[22] that when the variability in errors of observation are small relative to the range of the independent variable, as here, the problem of observation error is of no great concern.

Given that OLS is an adequate estimator of slope, it has several other advantages over the alternatives. First, and most importantly, OLS allows consideration of more complex models and the theory of least squares permits extensions such as Generalized Least Squares (GLS), which is useful in models incorporating phylogenetic information (see below). OLS is also consistent across different methods of assessing Rensch's rule [10]. For example, the results of a regression of log(female size) on log(male size) are statistically identical to those of a regression of SSD (log(female/male size)) on log(male size), a situation in which RMA produces nonsensical results (it cannot be used to test the H_0_ of a slope of 0) [10]. A further consideration is that the OLS slope will always be less than the RMA slope (but with an identical standard error [20]), and so rejecting Rensch's rule using OLS is more conservative than using RMA when log(female size) is regressed on log(male size) (i.e. when Rensch's rule predicts a slope <1).

A critical decision is the taxonomic level at which appropriate comparisons can be made. Previous analyses have varied considerably from populations within a species [11] to species within a family [15], [16], order [5], [23], or some combination of taxonomic levels [2], [3], [6]. Studies of higher taxa have often included only a small subset of all species within that taxon. Clearly, considering higher taxa will increase the number of species in an analysis, but may cause important within-taxon variation to be obscured [23]; alternatively, results may be overly influenced by differences between constituent subtaxa [13].

We consider species within families as our primary level of analysis. This decision is based partly on pragmatic grounds: there are 30 families in our dataset for which we have data for at least 10 species, enabling meaningful within-family analyses; together these families contain 1291 species (85% of the total). In addition, species within the same family are generally rather similar in terms of their ecology and life history. Importantly, patterns of SSD within families are reasonably consistent (in 19 of the 30 families that we analyse, at least 75% of the species have similar (i.e., MB or FB) SSD), and a significant fraction (48%) of total variation in SSD occurs between families (Anova, F_29, 1261_ = 40.3, *P* < 0.00001). To test whether including family as a factor in our analyses was sufficient to account for the phylogenetic structure in SSD in our dataset, we employed the method introduced by [24], developed by [25], and previously employed in a study of SSD by [5]. This involves estimating a parameter λ, which varies between 0 (indicating a trait which evolves independently of phylogeny) to 1 (the trait evolves in accordance with a Brownian motion model of evolution; see [25] for more details). We used Sibley and Ahlquist's DNA-DNA hybridization phylogeny [26] in this analysis. Whilst there are valid concerns regarding the methodology used by Sibley and Ahlquist, and the resulting tree topology, it remains the only phylogeny across the majority of bird families to include branch lengths [27] and has been successfully employed in recent broad-scale comparative analyses across all bird taxa [27], [28]. We supplement the family-level phylogeny with taxonomic information. Specifically, we assume that congeneric species will be more similar to each other than confamilial species which are in different genera; this increased similarity is scaled relative to the family-level branch length in each case.

We estimated λ for SSD across the 1291 species in the 30 families of interest. SSD is highly phylogenetically conserved, with a maximum likelihood (ML) value of λ of 1.0 which is significantly different from 0 (*P*<0.0001). However, including family as a factor in a model of log(female) on log(male) size (together with the interaction between log(male size) and family to allow for differences in slope between families) was sufficient to remove any phylogenetic structure from the residuals of the model: the ML value of λ from the phylogenetically weighted GLS model was 0, (significantly different from 1; *P*<0.00001). Setting λ to this ML value results in regression coefficients identical to OLS estimates, and so in the analyses below we employ OLS models which include family as a factor. Our results are not greatly affected by the way in which phylogeny is incorporated into the analysis: setting λ = 1 in a phylogenetically-weighted GLS model also results in identical parameter estimates. Likewise, varying tree topology below the family level has little effect on estimated slopes, and certainly our estimates do not appear to be biased (see Text S2 and Figure S2). We also explored the robustness of our results to variation within families by repeating the analysis at the genus level, which entailed a severe pruning of our dataset (only 19 genera, constituting 299 species, contained ≥ 10 species) but which produced qualitatively identical results (not shown). Likewise, including genus as a random factor in mixed effects models at the family level had little effect on the estimates of slope.

We used the above model to extract family-specific estimates of the slope of log(female size) on log(male size). We compared these family-specific slopes with the proportion of species in a family showing MBSSD (PMB). We compared a model in which PMB was treated as a continuous variable with one in which it was treated as a binary variable, dividing families into those with primarily (>50%) MBSSD and those with primarily (>50%) FBSSD. Finally, we compared family-specific slopes between those families with a PMB ≤0.2 and those with a PMB ≥0.8 (the proportions used to delimit taxa with FBSSD and MBSSD respectively in 2) to remove the issues surrounding tests of Rensch's rule in taxa with mixed SSD (see below). All linear models involving family-specific slopes as the response variable were weighted by the inverse of the standard error of the family-specific slope. Other measures of family-level SSD produce similar results. For instance, mean SSD is <1 in all families with a PMB >0.5, and >1 in all families with a PMB <0.5.

### Families with mixed SSD

As outlined above, assessments of Rensch's rule in taxa with mixed SSD risk confusing the alternative situations illustrated in figure 1. These alternatives can only be distinguished by considering separately the subsets of MB and FB species within families with mixed SSD. In some families, this involves a split along broadly taxonomic (i.e. evolutionary) grounds. For instance, in the Laridae, most gull species show MBSSD, whereas most terns, auks and skuas have FBSSD. In other groups, such a division makes sense on grounds of behaviour, life history and mechanisms of sexual selection. For instance, in shorebirds (Charadriiformes), males in species with FBSSD tend to perform agile aerial displays, favouring small male size, whereas males in species with MBSSD compete physically with each other and thus large size is selected for [5]. For all families with mixed SSD, we therefore estimate separate slopes for those species with MBSSD and those with FBSSD, and test whether the difference between these pairs of slopes differs significantly from 0. Separate slopes were only estimated for those 14 families with 0.2≤PMB≤0.8, and with at least 4 species with each kind of SSD. Finally, we compared all FBSSD slopes with all MBSSD slopes (i.e. two slopes from each family with mixed SSD and one from all families with a PMB of either ≤0.2 or ≥0.8), weighting the model by the reciprocal of the standard error of each slope estimate.

All statistical analyses were carried out in R [29].

### Comparison with other studies

We explored the generality of our results through a review of studies of scaling of SSD in a wide range of taxa with FBSSD, generally published since the two earlier reviews [2], [3]. We first estimated the slope of log(female) on log(male) size for the seven FBSSD taxa included in [2] by measuring slopes and 95% C.I.s from their figure 2. We also searched the literature for other studies of taxa with FBSSD which presented either a value for the slope of log(female) on log(male) size, or sufficient data to allow us to calculate such a slope. We considered studies of taxa with mixed SSD where data were available to allow us to analyse separately those species with FBSSD. All such re-analyses follow those described above. Where the data permitted, however, we did include a taxonomic variable (e.g. genus) as a factor in the model. In total we were able to obtain comparable estimates of slope for 28 distinct taxonomic groups, representing most major animal lineages in which FBSSD is observed and in which SSD has been investigated (arthropods, reptiles, fish and birds).

**Figure 2:**
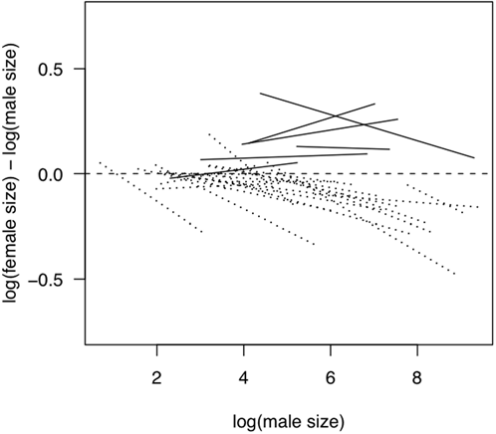
SSD (defined here as log(female size/male size)) against log(male size) for the 30 avian families in our dataset for which we had body size data for ≥ 10 species. The dashed horizontal line indicates no scaling of SSD with size. Also shown are OLS estimates of the relationship between SSD and log(male size) for each family; families with >50% FBSSD species are shown as solid lines, those with >50% MBSSD species as dotted lines. Note that this plot is equivalent to plotting log(female size) against log(male size) with the slopes presented in table 1, but it is easier to see differences between families on the SSD scale.

## Results

**Figure 3:**
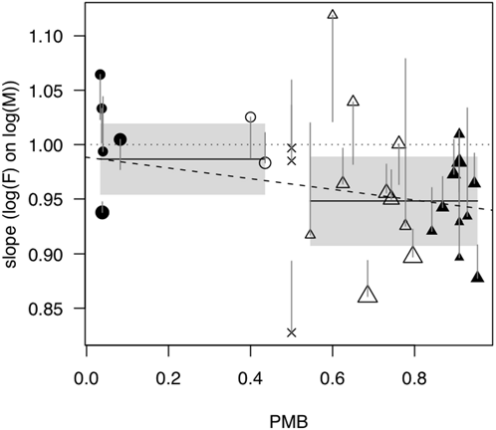
The family-specific slope estimates from table 1 plotted against PMB, the proportion of species within a family displaying MBSSD. The dotted horizontal line indicates isometry. Circles indicate families with PMB <0.5, triangles those with PMB >0.5, the horizontal solid lines are the means of these two groups, and the shaded grey regions are their respective 95% C.I.s. Solid symbols are those families with PMB ≤0.2 or ≥0.8. The size of each symbol is proportional to the number of species in that family for which we had data. Error bars represent the standard error of the family-specific slope estimates, and for clarity are extended only towards a slope of 1. Three families with a PMB of 0.5 are indicated by crosses, and were not included in the above analysis. Also shown (dashed line) is the simple linear regression of family-specific slope on PMB (weighted by the reciprocal of the s.e. of the family-specific slope).

The scaling of SSD with size varies significantly between families (family x log(male size) interaction: F_29, 1231_ = 4.79, *P*<0.00001). Family-specific slopes derived from this model are listed in table 1. In figure 2, we plot the same slopes derived from a model of SSD against log(male size) for increased visual clarity; note that the slopes in table 1 and figure 2 are statistically identical, differing only by a constant 1. Finally, family-specific slope estimates are plotted against the proportion of species in each family with MBSSD (PMB) in figure 3. Treating PMB as a continuous variable, the significant regression of family-specific slope (weighted by the inverse of its standard error) on PMB (dashed line in figure 3) has an intercept of 0.99 (95% C.I.: 0.95–1.03), which indicates a predicted slope indistinguishable from 1 in a family with exclusively FBSSD. The predicted slope for a family with exclusively MBSSD is significantly less than 1 (0.94, 95% C.I.: 0.90–0.97).

Dividing families into those with a PMB<0.5 and those with a PMB >0.5 resulted in 7 FBSSD and 20 MBSSD families (3 families with PMB = 0.5 excluded). Families with primarily FBSSD have slopes which do not differ significantly from 1 (0.99, 95% C.I.: 0.95–1.02), and families with primarily MBSSD have slopes significantly less than 1 (0.95, 95% C.I.: 0.90–0.99; shaded regions, figure 3). To eliminate the influence of families with mixed SSD, which can confuse the situations illustrated in figures 1A and B, we also consider only those families which display consistent MBSSD (PMB≥0.8, n = 10) or FBSSD (PMB≤0.2, n = 6), identified by filled symbols in figure 3. Again, the slope for families with FBSSD (1.04, 95% C.I.: 1.00–1.08) is indistinguishable from 1, whereas that for families with MBSSD is significantly less than 1 (0.93, 95% C.I.: 0.88–0.99).

### Families with mixed SSD

**Figure 4:**
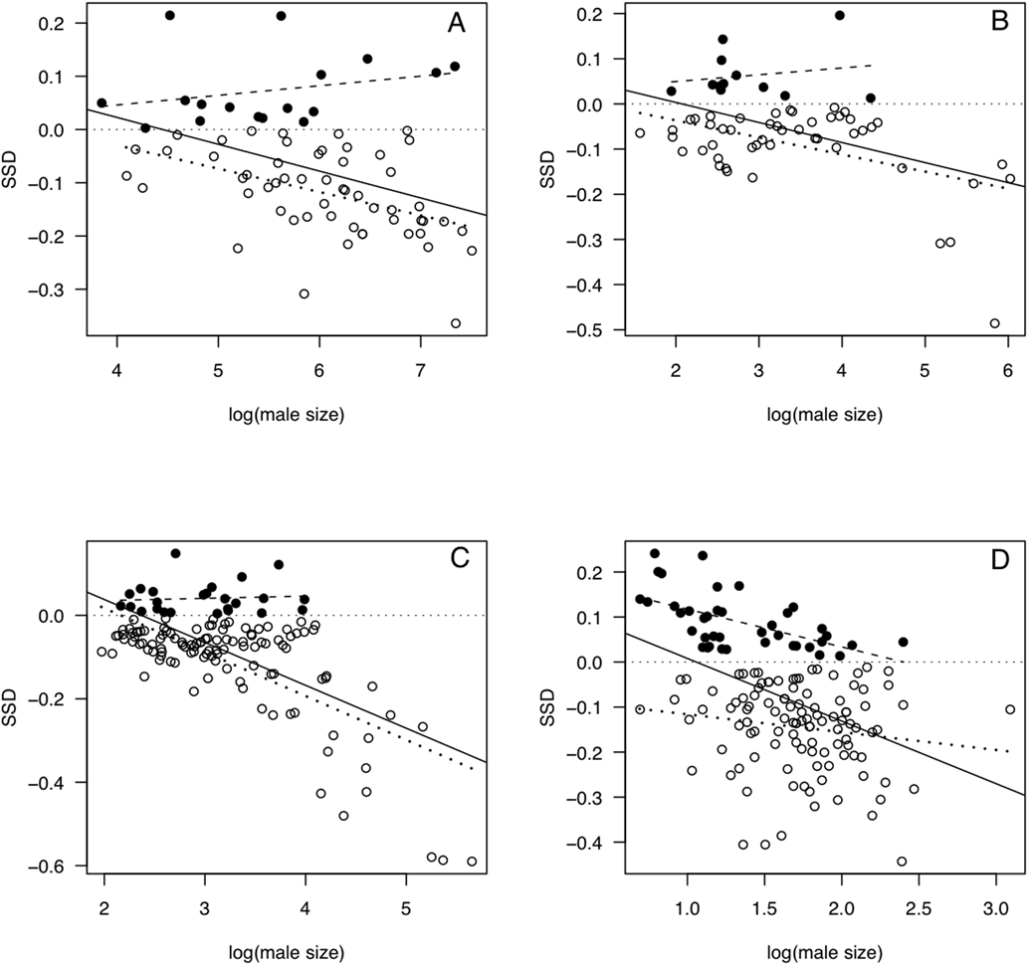
SSD against log(male size) for four families with mixed SSD, A. Laridae, B. Tyrannidae, C. Fringillidae, D. Trochilidae. In each case, species with FBSSD are shown as filled symbols, those with MBSSD as open symbols. We also include three regression lines on each figure. The solid line is the slope across all species, and is significantly negative in each case. The dashed line is the slope for species with FBSSD only, and differs from isometry only in D. The dotted line is for species with MBSSD only, which is significantly negative in all panels except D.

In the 14 families for which we were able to obtain separate estimates of slope for species with FBSSD and for those with MBSSD, slopes across the former group averaged 1.01 (95% C.I.: 0.98–1.03), whereas those across species with MBSSD averaged 0.95 (95% C.I.: 0.92–0.99). Typical families are illustrated in figure 4A–C, with an atypical family shown in figure 4D. The mean difference between the two slopes for each family was significantly different from 0 (0.05, 95% C.I.: 0.01–0.09; paired t = 2.83, d.f. = 13, *P* = 0.0142), indicating that on average the slope for those species within a family with FBSSD exceeded that for those species with MBSSD. The magnitude of these slopes did not vary systematically with the number of species involved in their estimation. Including MBSSD slopes from families with a PMB ≥0.8 and FBSSD slopes from families with a PMB ≤0.2 supports the previous results (mean slope, FBSSD species: 1.01, 95% C.I.: 0.98–1.03; mean slope, MBSSD species: 0.94, 95% C.I.: 91–97; difference between means, F_1, 42_ = 19.7, *P* = 0.00001; all means, C.I.s and tests weighted by 1/s.e. of the slope estimates).

### Other studies

Table 2 summarises the findings of interspecific studies of scaling of SSD in taxa with primarily FBSSD (as well as some taxa with mixed SSD for which data for species with FBSSD and MBSSD were available separately). There are several instances in table 2 where the authors of the study concluded that their results supported Rensch's rule, but we disagree. In some cases (e.g. Ascidae, Anispotera and Zygoptera, Charadriiformes) this is because the original analyses were of taxa with mixed SSD; when we re-analysed data only for species with FBSSD we did not find evidence of significant allometry in SSD. The discrepancy therefore rests on a difference of definition, with figure 1A rather than figure 1B supported. (The fact that in both the Charadriiformes and the Ascidae slopes <1 are found for those species with MBSSD actually suggests a subtly different situation, similar to that illustrated in figure 4A–C.) The support for the generality of Rensch's rule in [2] was based on a meta-analysis across 21 taxa with MBSSD, FBSSD or mixed SSD; we present only the 7 taxa with FBSSD in table 2. Grouping all 21 taxa, Abouheif and Fairbairn [2] considered owls (with a slope significantly >1; table 2) to be an outlier; excluding this taxon, they found homogeneous effect sizes across the remaining taxa and concluded that their results revealed “a highly significant, general trend consistent with Rensch's rule” (p 552). Considering only those taxa with FBSSD, however, we suggest that the owls (slope = 1.09) are far from an outlying taxon, as both elapid (1.09) and colubrid snakes (1.08) show very similar patterns, with raptors (1.00), sandpipers (0.98) and spiders (0.98) also closer to the owls than to the water striders (0.86), the only group with a slope significantly less than 1. These results are more consistent with the statement in [2] that “allometry for SSD is weak and variable in taxa in which females are the larger sex” (p 545).

**Table 2:** A summary of relationships obtained from the literature between log(male) and log(female) size from a wide range of taxa with predominantly FBSSD or with mixed MBSSD and FBSSD in which data were provided separately for FBSSD and MBSSD species.

Taxon	N^a^	Slope	Rensch's rule supported?^b^		Source
			by the authors	by us	
***Arthropods***					
Spiders (Araneomorphae)	44 (30000); ≥36^c^	0.98 (0.74–1.22)^d^	Y^e^	N	2
Spiders (Araneomorphae)	13 (30000); 13^f^	0.88±0.135	Y	N	6
Orb-weaving spiders (Orbiculariae)	602 (10000); 579^f^	0.99±0.017	-	N	40
Beetles (Coleoptera)	58 (>350 000); 52	1.01±0.013	N	N	6
Ladybird beetles (Coccinellidae)	37 (5000); 37	1.00±0.017	N	N	6
Hummingbird flower mites (Ascidae)	37 (70); 21	0.96±0.042	Y	N	12
Water striders (Gerridae)	46 (500); ≥37^c^	0.86 (0.80–0.91)^d^	Y^e^	Y	2
Water striders (Gerridae)	112 (500); 93	0.93±0.020	Y	Y	41^g^
Dragonflies (Anisoptera and Zygoptera)	21 (5572); 11	0.97±0.013	Y	N	13
Tephritid flies (Tephritidae)^h^	32 (4352); 24	0.91±0.019^i^	Y	Y	34
Fruit flies (Drosophilidae)	72 (4000); 72	0.81±0.027	Y	Y	6, 42, 43^i^
Diopsid flies (Diopsidae)	30 (200); 26	0.88±0.024	Y	Y	44
Sepsid flies (Sepsidae)	10 (250); 8	0.99±0.107	Y	N	6
N. American Hydrapsychid caddisflies	29 (144); 27	1.05±0.043	N	N	45
Hymenoptera	20 (115 000); 19	0.98±0.017	N	N	6
Lepidoptera	47 (140 000); 37	0.92±0.039	Y	N^j^	6
Stick insects (Phasmatodea)	152 (2500); ?	0.84^k^	-	-	34
***Reptiles***					
Eublepharid geckos (Eublepharidae)	11 (20); 5	0.97±0.032	Y	N	15
Colubrid snakes (Colubridae)	18 (1800); ≥15^c^	1.08 (0.95–1.21)^d^	Y^e^	N	2
Australian Elapid snakes (Elapidae)	19 (77); ≥16^c^	1.09 (0.64–1.54)^d^	Y^e^	N	2
***Fish***					
Sharks and rays (Chondrithyes)	102 (890); 86	1.00±0.020	N	N	Webb, Dulvy & Freckleton, unpublished analysis
North American minnows (Cyprinidae)	61 (411); 28	0.98±0.022	-	N	30
***Birds***					
Tinamous (Tinamidae)^j^	25 (47); 24	1.00±0.020	N	N	17
Hummingbirds (Trochilidae)	154 (330); 36	0.92±0.022	Y	Y	12
Raptors (Falconiformes)	22 (306); ≥18^c^	1.00 (0.85–1.14)^d^	Y^e^	N	2
Owls (Strigiformes)	25 (189); ≥20^c^	1.09 (0.98–1.20)^d^	Y^e^	N	2
Sandpipers (Scolopacidae)	35 (86); ≥28^c^	0.98 (0.83–1.14)^d^	Y^e^	N	2
Shorebirds (Ciconiiformes)	102 (345); 47	0.99±0.016	Y	N	5

a The three values for N are: the number of species in the study (approximate number of species in the taxonomic or functional group considered); number of species in sample with FBSSD.

b We expand upon our reasons for any disagreement in the text.

c Taxa are classified in [2] as female-biased if at least 80% of species in the sample had FBSSD.

d Estimates of the slope and its 95% C.I. were measured off figure 2 of [2].

e The conclusion in [2] that Rensch's rule was a general phenomenon based on a meta-analysis of the taxa listed here as well as taxa with MBSSD and mixed SSD, and so we list them as supporting Rensch's rule in each case; whereas we consider each taxon separately.

f Sample sizes given are for all species in which females are larger than males. However, in the analysis we consider only those species in which the female is ≤2 x the size of the male, which is the conventional delimitation of ‘extreme’ SSD in spiders [40]. When females are much larger than males in this group, the correlation between male and female size breaks down, and it is not clear what the appropriate technique for examining scaling in SSD is in such a situation. The sample sizes for the analyses using data from [6] and [40] were therefore 11 and 476 species respectively.

g We analyse data for all species-level data given in the appendix to [41]; note that the analyses presented [41] are at the subfamily level, and use data for 209 species of waterstrider. He found evidence for Rensch's rule in 8 of 9 subfamilies with at least 10 species; we too support Rensch's rule for species with FBSSD in the two largest subfamilies, Gerrinae (n = 59, slope = 0.89±0.028, *P* = 0.0003) and Halobatinae (n = 21, slope = 0.78±0.081, *P* = 0.0144).

h We focus on thorax length from the several morphological measures given in [34].

i We have combined data from the three sources cited in the table. For the small number of species occurring in more than one dataset, we took the arithmetic mean size (across datasets) for male and female size. Including source as a factor in the analysis did not substantially affect the estimate of the slope (common slope estimated as 0.86±0.028, significantly <1 with *P*<0.00001).

j We reject Rensch's rule in this case as the *P* value for the test for a slope significantly different from 1 is 0.0553, but accept that this is only marginally non-significant.

k This slope is an unpublished result mentioned in [34], and is the slope of log(male) on log(female) size, i.e. a situation in which Rensch's rule predicts a slope >1.

l We focus on body mass from the several morphological measures given in [17].

Allometry consistent with Rensch's rule occurs most frequently in arthropods, although even here it is supported in only 5 of 17 studies. The strongest evidence comes from tephritid, diopsid and fruit flies, and from water striders, with no significant departures from a slope of 1 in any of the other taxa examined. We note that the arthropod studies typically encompass only a tiny proportion of the total number of described species in each group (median = 1.8%), often <10% of known species. However, it is not clear how this would introduce any kind of bias, and contrasting patterns are observed even across the more thoroughly sampled groups (e.g. waterstriders and North American caddisflies, both including around 20% of described species; table 2). In addition, the species included in a study will seldom be a random sample of all those described, and may in fact constitute a much larger proportion of a well-defined functional group, often in a particular geographic area, so the figures presented in table 2 should be seen as minimum proportions.

Taxonomic issues confound some studies. For instance, the study of Odonata [13] included species from two suborders; including suborder as a factor in the analysis changes the slope across species with FBSSD from significantly less than 1 (0.97±0.013; table 2) to indistinguishable from 1 (0.99±0.014). Note that this strong phylogenetic effect–a single large difference between suborders–was not identified by the use in [13] of independent contrasts, which are not designed to control for grade shifts between clades.

The data we obtained from the study of minnows [30] provide another instructive example: slopes differ between genera depending on their prevalent form of SSD. Thus, in *Cyprinella* 13 of 14 species show MBSSD and the slope is significantly <1 (0.79±0.087, t = 2.45, *P* = 0.0305); a similar pattern occurs in *Hybopsis* (7 of 11 species show MBSSD; slope = 0.72±0.133, t = 2.15, *P* = 0.0603) but a slope indistinguishable from 1 is observed in *Notropis* (3 of 11 species show MBSSD; slope = 1.06±0.080, t = 0.75, *P* = 0.463). This analysis reaffirms that our methods are capable of detecting allometry consistent with Rensch's rule where it does occur (i.e. in taxa with predominantly MBSSD), and that these methods are capable of offering more insight into the structure of such patterns than cross-species analysis, or the method of independent contrasts commonly employed in previous studies of Rensch's rule.

## Discussion

Our results confirm that, across bird families with primarily MBSSD, SSD scales positively with male size in accordance with Rensch's rule, as is generally the case in taxa with MBSSD [2], [3], [18]. However, we find no evidence that Rensch's rule applies as a general phenomenon across avian families with primarily FBSSD, despite the fact that by estimating slopes using OLS, our results are biased towards acceptance of Rensch's rule. These findings are in broad accordance with those from previous reviews of Rensch's rule [2], [3], [6], but disagree with the way that this evidence continues to be presented (i.e., that Rensch's rule is a general and consistent phenomenon).

Our work underlines that Rensch's rule is sensitive to taxonomic level, as suggested by [23]. These authors showed that overall support for Rensch's rule across primates was somewhat misleading, as the rule does not apply to prosimians; likewise, the Haplorhini (in which the rule was supported) could further be divided into the Old World monkeys and apes (Catarrhini), which show Rensch's rule, and the New World monkeys (Platyrrhini) which do not [23]. Other studies [2] have attempted to address this issue by successively grouping subtaxa together which did not show heterogeneity of slopes. This approach is critically dependent on sample size, however. For instance, Abouheif and Fairbairn [2] grouped together all raptors for which they had data (n = 21), with a resulting slope of 1.00. Had our analysis, incorporating data on substantially more raptor species (n = 134), considered this group together we would have concluded that SSD scaled negatively with size (slope = 0.96±0.013, t = 3.34, *P* = 0.0011); but in fact the pattern in the Falconidae (slope = 1.06±0.032, n = 30) is different from that in the Accipitridae (0.94±0.015, n = 104). Moreover, the approach of grouping taxa with statistically indistinguishable slopes confounds statistical testing (i.e. testing for differences in slopes) with estimation (using the results of these tests to influence parameter estimates), with the consequence that estimates of the slopes of relationships may be biased [31].

The issue of which taxonomic level is appropriate for analysis is thus complicated, and may depend both on data availability and on the particular evolutionary hypothesis being addressed. However, we suggest that the use of modern comparative methods, for instance using GLS, estimating λ or using hierarchical models, can provide useful information both about the taxonomic level at which most variation in SSD resides, and the adequacy of including taxonomic information to account for the phylogenetic structure of the data.

The quality of data used in comparative analyses of body size is seldom given the attention it deserves [32]. For instance, of the studies listed in table 2, one of the few examples of SSD scaling with size in a manner consistent with Rensch's rule occurs in 36 hummingbird species with FBSSD (see also figure 4D), yet in only four of these species was the sample size >1 for both sexes [12]. This study is unusual among studies of birds only in that sample size is reported; this issue surely affects most other studies across large numbers of species. Although slope estimates for individual families may be somewhat affected by this issue, it is difficult to see how it could introduce any systematic bias to produce the general result that we find here, i.e. that (with rare exceptions) Rensch's rule is only supported in taxa with predominantly MBSSD.

Attempts to explain Rensch's rule as a general phenomenon have generated large numbers of competing hypotheses (reviewed in 3); as Fairbairn (14: 570) states, “The existence of allometry consistent with Rensch's rule as a common, repeatable evolutionary pattern prompts a search for a common functional explanation”. However, accepting that Rensch's rule is a common, repeatable evolutionary phenomenon only in taxa in which males are larger than females suggests that such searches for common, functional explanations across taxa may be misguided. In taxa with MBSSD, hypotheses based on sexual selection on male size have been effective at explaining Rensch's rule [5], [16], [33]; such hypotheses are generally less convincing for species with FBSSD [14], and may require rather convoluted evolutionary scenarios (e.g. sexual selection for large males would explain Rensch's rule in taxa with FBSSD *if* these taxa evolved from a smaller, more sexually dimorphic ancestor; 11). This is not to say that such processes have not occurred from time to time, and there is good support for particular mechanisms of sexual selection generating patterns consistent with Rensch's rule in certain groups of birds with FB or mixed SSD. For instance, sexual selection for small male size is sometimes predicted, particularly in smaller species. This may result from direct selection for agile courtship displays (5; similar effects may occur in many invertebrates [34] as well as in other vertebrate taxa including bats and primates) or from different strategies evolving for coping with the costs of energetic courtship displays (males in resource-rich environments become larger, those in resource-poor environments become smaller) [12]. As an example, in red knot *Calidris canutus* (a shorebird) females are c. 15% larger than males, and males compete for females by performing elaborate display flights [35].

Several alternative evolutionary routes to FBSSD have been proposed for birds [36], including sexual selection for large female size in species with reversed sexual roles. For example in the red-necked phalarope *Phalaropus lobatus*, another shorebird with a similar degree of SSD to the knot, sex roles are reversed and females compete aggressively for males [35]. Still other evolutionary pathways to FBSSD are possible, even within the same taxonomic group, for example selection for female dominance over males to facilitate pair bonding in birds with raptorial lifestyles in the shorebird family Stercorariidae [37], [see also 36]. ‘Agility’ based hypotheses may prove effective at explaining why species with FBSSD are often smaller than species with MBSSD in groups with mixed SSD (figure 1A), but it is harder to see how they might explain a decline in SSD with increasing size in groups with exclusively FBSSD (i.e. Rensch's rule; figure 1B), and given that FBSSD can result from rather different selective regimes, even within quite closely related taxa, it is perhaps not surprising that consistent patterns of allometry for SSD in taxa with FBSSD are not observed. This is in contrast to the relatively high explanatory power of simple explanations for the evolution of MBSSD based on sexual selection, where including some measure of the strength of male-male competition can often explain most of the variation in SSD, independent of male size [5], [16], [33], perhaps due to scaling of secondary sexual traits [38] as originally proposed by Rensch [8].

In conclusion, we have confirmed that Rensch's rule is well supported in birds with MBSSD. However, there is no evidence from our dataset, and very little from the literature, for Rensch's contention [8] that SSD decreases with increasing body size in taxa with FBSSD as a general rule. There are exceptions, but these have usually been explained in terms of specific and presumably rather unusual sets of circumstances. When only exceptional cases provide the supporting evidence, we would question the generality of a ‘rule’. Evolutionary explanations for scaling of SSD with size in those taxa with FBSSD which do support Rensch's rule (e.g. water striders) [11] are therefore unlikely to generalise to other taxa with FBSSD. In general, and unlike more universal allometric relationships in ecology [39], Rensch's rule does not appear to exist as an independent scaling phenomenon; allometric scaling in SSD can usually be explained by incorporating one or two explanatory variables (in addition to body size) into an analysis [5], [16]. Clearly further work is required to work out what factors affect FBSSD.

## Supporting Information

Text S1 The effects of error in the independent variable: comparing OLS, RMA and SIMEX estimates of the slope of log(female size) on log(male size)
(0.02 MB DOC)

Text S2 The effects of varying intra-familial phylogeny on estimates of the slope of log(female size) on log(male size)
(0.04 MB DOC)

Figure S1 SIMEX estimation of the slope of log(female size) on log(male size) for A. primates and B. Hydropsychid caddisflies. Open circles are estimates of the slope including various amounts of simulated variation, the solid line represents extrapolation using a GAM (the triangle at variance = 0 is the resulting SIMEX slope estimate) and the dashed line is a linear extrapolation (SIMEX slope estimate is a cross). The OLS (solid circle) and RMA (diamond) estimates are also shown.
(0.07 MB TIF)

Figure S2 Density plots of 100 slopes of log(female size) on log(male size) derived from PGLMs for Phasianidae (A, dashed line), Falconidae (A, solid line), Fringillidae species with MBSSD (B, dashed line) and Fringillidae species with FBSSD (B, solid line) using randomly generated phylogenies. In each case, the relevant OLS estimate is indicated with a vertical line.
(0.08 MB TIF)
