# Supplementary material for: Only Half Right: Species with Female-Biased Sexual Size Dimorphism Consistently Break Rensch's Rule
Source: PLoS One. 2007 Sep 19;2(9):e897. doi: 10.1371/journal.pone.0000897 (PMC1964802; doi:10.1371/journal.pone.0000897)
Supplement: Text S1 — The effects of error in the independent variable: comparing OLS, RMA and SIMEX estimates of the slope of log(female size) on log(male size) (0.02 MB DOC) [file pone.0000897.s001.doc]

***Webb & Freckleton, Rensch’s rule and female-biased SSD, Text S1***

***The effects of error in the independent variable: comparing OLS, RMA and SIMEX estimates of the slope of log(female size) on log(male size)***

We employ the SIMEX approach described in (1) as implemented in (2) to estimate the impact of intraspecific variation in male size (the independent variable) on the slope of the relationship of female on male size for two datasets for which we have information on intraspecific variance in male size. The first dataset, taken from (3), includes the mean and variance in male and female mass for 19 species of primate, with each mean and variance based on the measurement of between 17 and 502 individuals. The second dataset, taken from (4) is of mean and variance in male and female body length across 29 species of North American Hydropsychid caddisflies (Trichoptera), based on measurements of between 9 and 40 individuals.

The simulation part of the SIMEX procedure involves adding increasing amounts of pseudo-error to the independent variable (log(male size) in our case), and repeatedly calculating the OLS slope of log(female) on log(male) size with these differing degrees of pseudo-error. At each level of added variation, the pseudo-error for a given species was proportional to the observed variance in body size within that species. We calculated mean values of the slope of the log(female size) on log(male size) relationship (termed *beta*) across 1000 iterations of each multiple of the observed variance. Note that variance = 0.5 is the OLS estimate of the slope. The extrapolation then proceeds by fitting a model to the relationship between beta and variance, and then extrapolating this back to 0 to give an estimate of the value of beta with no error in the measurement of male size. We employ two models on which to base the extrapolation, a simple linear model of the form beta ~ variance, and a non-parametric smoothed function fitted as a Generalized Additive Model of the form beta ~ *s*(variance). In this latter case, we use the *mgcv* package in R (5, 6) to select the optimal smoothing function *s* using generalized cross validation.

Results are shown in figure S1. In the primates (figure S1A), the simple OLS estimate of the slope of log(female) on log(male) mass was 0.936, whereas the RMA estimate was 0.941. The SIMEX estimate of the slope was 0.937 for both the linear and the GAM extrapolation functions, although the GAM appeared to fit the data better (R^2^ = 0.96 *cf*. 0.79 in the linear model). In the caddisflies (figure S1B), the simple OLS estimate of the slope of log(female) on log(male) length was 1.056, and the RMA slope was 1.082. The SIMEX slope estimate was 1.060 using the GAM extrapolation function, and 1.074 using the linear. The GAM function fits the observed data substantially better (R^2^ = 0.99 *cf.* 0.87 for the linear). In both the primates and the caddisflies then, the SIMEX approach suggests that the OLS estimate of the relationship between male and female size is marginally preferable to the RMA estimate. In both cases, the maximum difference occurred between RMA and OLS slopes, yet even these differed by less than 2.5%.

**References**

1 Cook, JR, Stefanski, LA (1994) Simulation-Extrapolation estimation in parametric measurement error models. J Am Stat Assoc 89: 1314-1328.

2 Faraway, J (2004) Linear models with R. Boca Raton, FL: Chapman & Hall/CRC.

3 Smith, RJ, Jungers, WL (1997) Body mass in comparative primatology. J Hum Evol 32: 523-559.

4 Jannot, JE, Kerans, BL (2003) Body size, sexual size dimorphism, and Rensch's rule in adult hydropsychid caddisflies (Trichoptera: Hydropsychidae). Can J Zool 81: 1956-1964.

5 R Development Core Team (2006) R: A Language and Environment for Statistical Computing. Vienna, Austria: R Foundation for Statistical Computing. http://www.R-project.org

6 Wood, SN (2006) Generalized Additive Models: an Introduction with R. Boca Raton, FL: Chapman and Hall/CRC.
