# Supplementary material for: Only Half Right: Species with Female-Biased Sexual Size Dimorphism Consistently Break Rensch's Rule
Source: PLoS One. 2007 Sep 19;2(9):e897. doi: 10.1371/journal.pone.0000897 (PMC1964802; doi:10.1371/journal.pone.0000897)
Supplement: Text S2 — The effects of varying intra-familial phylogeny on estimates of the slope of log(female size) on log(male size) (0.04 MB DOC) [file pone.0000897.s002.doc]

***Webb & Freckleton, Rensch’s rule and female-biased SSD, Text S2***

***The effects of varying intra-familial phylogeny on estimates of the slope of log(female size) on log(male size)***

For some families, it is possible to use detailed species-level phylogenies to explore the effects of phylogeny below the level of the family on our results. For instance, we obtained phylogenies for 52 species in the genera *Larus*, *Pagophila*, *Rhodostethia*, *Rissa*, and *Xema* in the family Laridae (A. Phillimore, unpublished data). Briefly, Cytochrome b sequences obtained from Genbank were used to reconstruct phylogenies using a relaxed clock Bayesian method (1) implemented in Beast (2, 3), which employs an MCMC approach. Two runs of 5 million generations were conducted, which, after excluding 1 million burn-in generations in each run, resulted in 2000 trees from the posterior distribution of the runs, sampled as one every 4000 generations. We used a sample of 1000 of these trees, pruned to the 32 species for which we had male and female body size data. For each tree, we ran a phylogenetically-weighted generalized least squares model (PGLM) with λ set to 1 (PGLM-1), and one PGLM with λ taking its maximum likelihood value (PGLM-ML). All models are of the form: log(female size) ~ log(male size). The mean value of the slope estimate from PGLM-1 models was 0.936, with an empirical 95% C.I. of 0.894 – 0.996. For the PGLM-ML models, the mean slope was 0.930 (0.912 – 0.946). This compares with an OLS estimate of the slope of 0.935 (calculated 95% C.I.: 0.906 – 0.965) and an RMA slope of 0.939 (0.909 – 0.968).

The distribution of ML estimates of λ was strongly bimodal: more than half the estimates (512) exceeded 0.95, but a further 28% (277) were less than 0.0001. This explains the wider C.I. for PGLMs forcing λ to equal 1 (and the range of values, 0.848–1.066, compared to 0.895–0.967 for the ML approach), and shows the advantage of using an optimised degree of phylogenetic correction, as opposed to the ‘all or nothing’ approach of some other methods.

In general, both the OLS estimate and the RMA estimate provide good approximations of the estimates derived using the PGLM-ML. With male and female size so strongly correlated, it is perhaps not surprising that sub-familial phylogeny has little impact on the estimates of slope.

We do not have species-level phylogenies similar to that for Laridae for many groups in our dataset. However, we can get some idea of how sensitive our results are to sub-familial phylogeny by generating a random phylogeny, and fitting a PGLM assuming a λ of 1 (i.e. a very strong phylogenetic signal in SSD). This will show the degree to which the slope of log(female) on log(male) size can vary with different phylogenetic hypotheses, and can be used to assess the adequacy of the OLS estimate.

We illustrate this for two families drawn from the extremes of our dataset: Falconidae (n = 30 species), where almost all species have FBSSD, and Phasianidae (n = 43 species), where the opposite is true. We also consider the Fringillidae as an example of a family with mixed SSD (n = 25 species with FBSSD, 113 species with MBSSD). For each family, we generated a set of 100 random phylogenies using the function *rcoal* in the *ape* package in R (4, 5). For each phylogeny, we estimate the slope of the relationship between log(male) and log(female) size, using a PGLM with λ = 1 (i.e. assuming strong phylogenetic signal in the data). For the Fringillidae, we include SSD (MB or FB) as a factor, together with its interaction with log(male size), to give separate estimates of slope for species with MBSSD and those with FBSSD for each iteration of the phylogeny.

Results are shown in figure S2. For all families, the mean slopes from the PGLMs were almost identical to the OLS estimate (Falconidae, OLS = 1.06, mean ± s.d. PGLM = 1.06 ± 0.092; Phasianidae, OLS = 0.88, PGLM = 0.88 ± 0.078; Fringillidae, FBSSD, OLS = 1.01, PGLM = 1.00 ± 0.043; Fringillidae, MBSSD, OLS = 0.89, PGLM = 0.90 ± 0.037). For the Fringillidae, the slope for species with FBSSD was greater than that for species with MBSSD on 96 occasions, by a mean of 0.10 (*cf.* a difference in slopes of 0.11 estimated by OLS).

Clearly, sub-familial phylogeny can have an effect on the estimated slope of the relationship (see the range of values depicted in each case in figure S2). However, even under the extreme situation illustrated here, in which we assume no knowledge about sub-familial relationships (whereas in our main analyses we considered generic relationships), and in which we force λ to equal 1 rather than to take its ML value, it is clear that OLS provides an unbiased estimate of the slope. Thus, although individual family-specific slopes in our main analysis may be affected by sub-familial phylogeny, the broad, general patterns we describe across all families cannot be an artefact of incorrect or insufficient phylogenetic information.

**References**

1 Drummond AJ, Ho SYW, Phillips MJ, Rambaut A (2006) Relaxed Phylogenetics and Dating with Confidence. PLoS Biology 4: e88.

2 Drummond AJ, Nicholls GK, Rodrigo AG, Solomon W (2002) Estimating Mutation Parameters, Population History and Genealogy Simultaneously From Temporally Spaced Sequence Data. Genetics 161: 1307-1320.

3 Drummond AJ, Rambaut A (2003) BEAST version 1.3 [computer program] Available: http://evolve.zoo.ox.ac.uk/beast. Accessed 31 January 2006

4 R Development Core Team (2006) R: A Language and Environment for Statistical Computing. Vienna, Austria: R Foundation for Statistical Computing. http://www.R-project.org

5 Paradis E, Claude J, Strimmer K (2004) APE: analyses of phylogenetics and evolution in R language. Bioinformatics 20: 289-290.
