# Supplementary figures and images for: Only Half Right: Species with Female-Biased Sexual Size Dimorphism Consistently Break Rensch's Rule
Source: PLoS One. 2007 Sep 19;2(9):e897. doi: 10.1371/journal.pone.0000897 (PMC1964802; doi:10.1371/journal.pone.0000897)

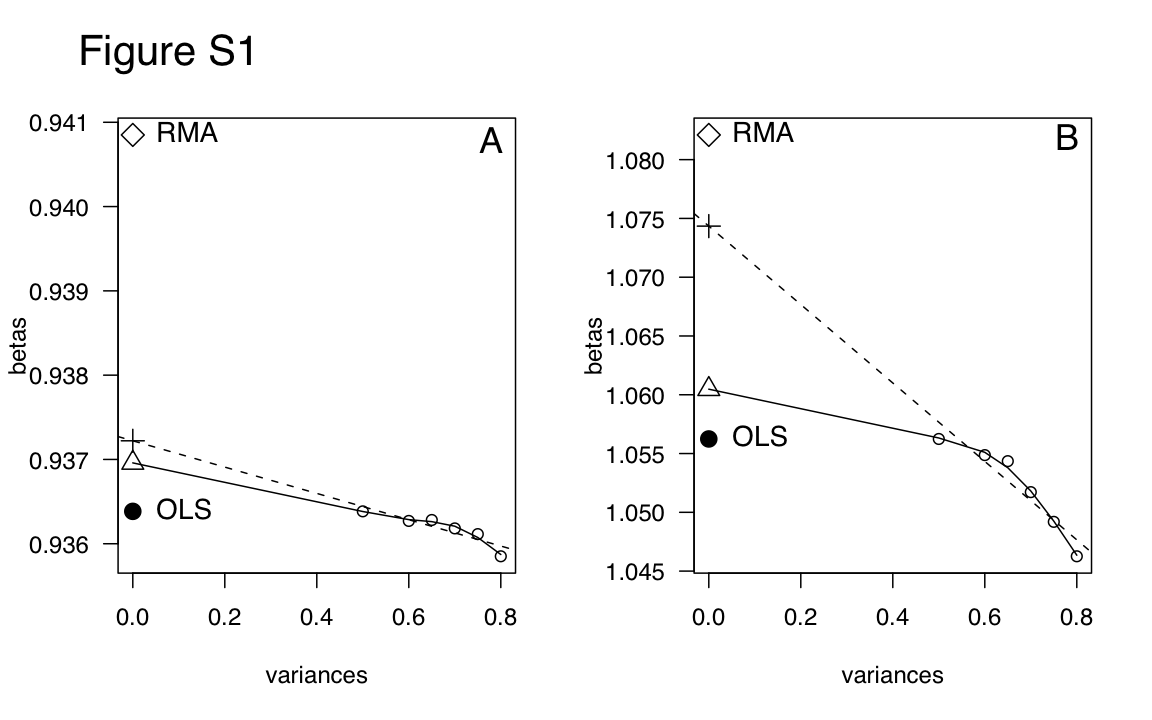

Supplement: Figure S1 — SIMEX estimation of the slope of log(female size) on log(male size) for A. primates and B. Hydropsychid caddisflies. Open circles are estimates of the slope including various amounts of simulated variation, the solid line represents extrapolation using a GAM (the triangle at variance = 0 is the resulting SIMEX slope estimate) and the dashed line is a linear extrapolation (SIMEX slope estimate is a cross). The OLS (solid circle) and RMA (diamond) estimates are also shown. (0.07 MB TIF) [file pone.0000897.s003.tif]

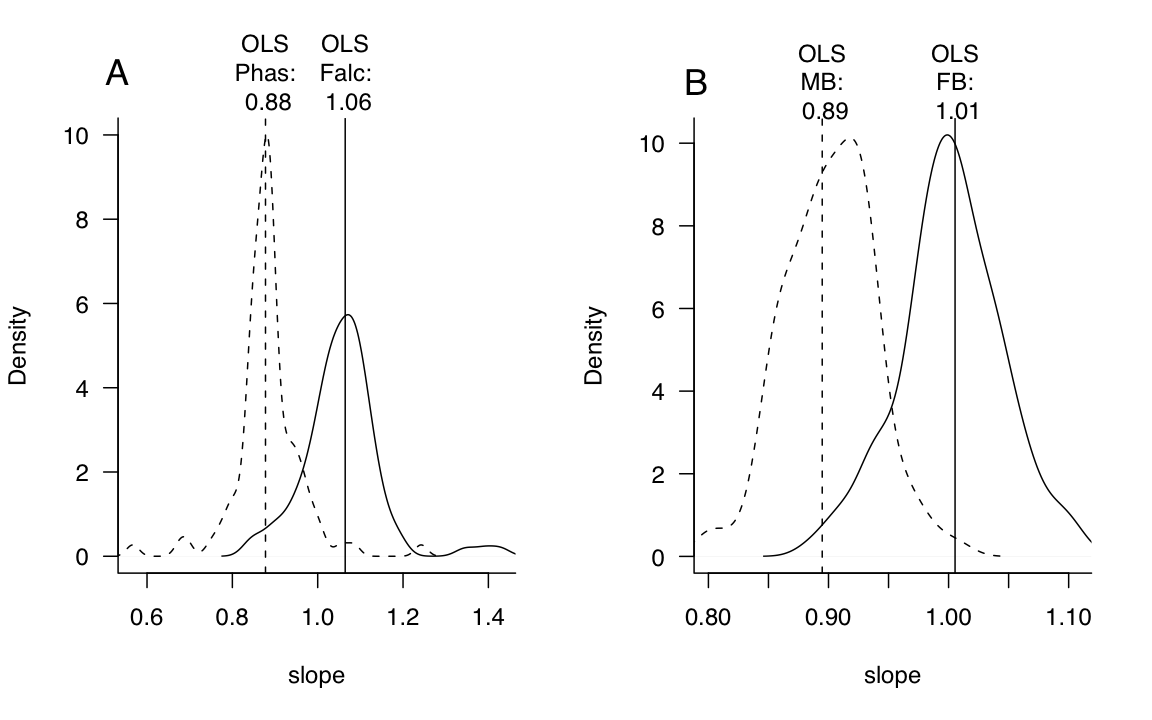

Supplement: Figure S2 — Density plots of 100 slopes of log(female size) on log(male size) derived from PGLMs for Phasianidae (A, dashed line), Falconidae (A, solid line), Fringillidae species with MBSSD (B, dashed line) and Fringillidae species with FBSSD (B, solid line) using randomly generated phylogenies. In each case, the relevant OLS estimate is indicated with a vertical line. (0.08 MB TIF) [file pone.0000897.s004.tif]
